# Refractory cavernous sinus thrombophlebitis complicated by brain abscess and infectious hydrocephalus: a case report and literature review

**DOI:** 10.1186/s12879-026-13208-7

**Published:** 2026-04-24

**Authors:** Yuanyuan Chen, Jiacheng Liu, Song Wang, Qingpei Hao, Xia Liu, Yan Gao

**Affiliations:** 1 Department of Infectious Diseases, Peking University Hepatology Institute, Peking University People’s Hospital, Beijing, People’s Republic of China; 2 Department of Endocrinology, Peking University People’s Hospital, Beijing, People’s Republic of China; 3 Department of Neurosurgery, Peking University People’s Hospital, Beijing, People’s Republic of China; 4 Department of Radiology, Peking University People’s Hospital, Beijing, People’s Republic of China

**Keywords:** Cavernous sinus thrombophlebitis, Brain abscess, Hydrocephalus, Staphylococcus aureus, Multidisciplinary team, Intraventricular antimicrobial therapy

## Abstract

**Background:**

Cavernous sinus thrombophlebitis (CST) is a rare, life-threatening infection. Its management becomes exceptionally challenging when complicated by intracranial abscesses, hydrocephalus, and multidrug-resistant pathogens such as methicillin-resistant *Staphylococcus aureus* (MRSA).

**Case presentation:**

A 52-year-old woman with poorly controlled diabetes presented with headache, eye pain, diplopia, fever, and progressive ocular symptoms. Outpatient ceftriaxone and subsequent ertapenem were ineffective. Neuroimaging confirmed CST with early brain abscess formation. Despite systemic antimicrobial therapy (meropenem, vancomycin, linezolid) and anticoagulation, she developed cerebral infarction, hydrocephalus requiring Ommaya reservoir placement, and persistent positive cerebrospinal fluid (CSF) cultures for MRSA and other organisms. Following multidisciplinary team (MDT) review involving infectious diseases, neurosurgery, ophthalmology, and otorhinolaryngology, intraventricular vancomycin (15 mg daily) was administered via an external ventricular drain, achieving CSF sterilization by day 79. A ventriculoperitoneal shunt was subsequently placed with sustained clinical improvement.

**Conclusions:**

This refractory MRSA-associated CST case illustrates the critical limitations of systemic antibiotics in penetrating the central nervous system. Successful outcome depended on: (1) early recognition in an at-risk host, (2) timely escalation to intraventricular therapy with a practical, evidence-based regimen, and (3) seamless MDT integration for diagnostic clarity and treatment sequencing. The detailed dosing and monitoring data provided here address a knowledge gap and offer a clinical template for managing similar complex CNS infections, with the potential for complete neurological recovery as demonstrated in this case.

## Introduction

Cavernous sinus thrombosis (CST) is a rare yet life-threatening infectious thrombophlebitis, with an estimated incidence of 0.2–1.6 per 100,000 individuals [1]. Although advancements in diagnosis and management have reduced its mortality to approximately 11%, associated morbidity remains considerable, with a disability rate of around 15% [2]. The clinical presentation typically features systemic signs of infection along with characteristic ocular manifestations, including ophthalmoplegia, proptosis, and visual impairment [3]. CST most commonly arises from contiguous spread of infections originating in the facial “danger triangle,” paranasal sinuses, or odontogenic foci [3, 4].

Non-infectious etiologies are relatively uncommon and may include malignancies (e.g., oral squamous cell carcinoma) [5], pharmacological factors (such as high-dose corticosteroid therapy) [6], surgical or traumatic events [7], and underlying hypercoagulable states [4].

Despite well-documented classic features, recognition of CST is often delayed. Management becomes substantially more challenging when CST is complicated by intracranial abscesses or infectious hydrocephalus, particularly when caused by multidrug-resistant organisms (MDROs) such as MRSA. Treatment of such complex cases necessitates close multidisciplinary collaboration; however, systematic strategies and shared clinical experiences regarding refractory, complicated CST remain inadequately represented in the literature.

This report presents a case of refractory MRSA-associated CST complicated by brain abscess and infectious hydrocephalus. We aim to detail the diagnostic challenges, multidisciplinary decision-making process, and clinical outcomes, with emphasis on practical insights regarding intraventricular antimicrobial therapy—an intervention often mentioned but rarely described with sufficient granularity to guide clinical practice.

## Case presentation

**Fig. 1:**
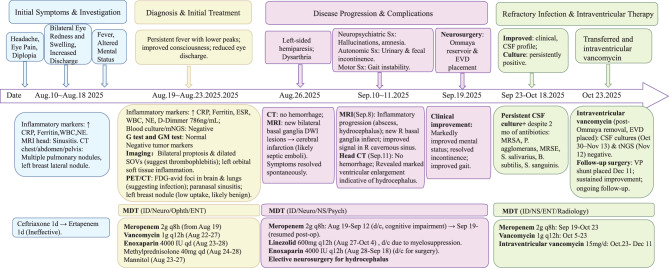
Clinical timeline and key interventions. A comprehensive flowchart summarizing the patient’s hospital course from symptom onset through final follow-up. The timeline integrates presenting symptoms, diagnostic findings (imaging, laboratory, microbiology), antimicrobial and adjunctive therapies, surgical procedures, and multidisciplinary team (MDT) involvement. Abbreviations: MDT, multidisciplinary team (infectious diseases, neurosurgery, ophthalmology, otorhinolaryngology); SOV, superior ophthalmic vein; mNGS, metagenomic next-generation sequencing; tNGS, targeted next-generation sequencing; MRSA, methicillin-resistant *Staphylococcus aureus*; MRSE, methicillin-resistant *Staphylococcus epidermidis*; EVD, external ventricular drain; VP, ventriculoperitoneal

A 52-year-old woman with poorly controlled diabetes presented with a 9-day history of headache and eye pain, and 5-day history of high-grade fever (Tmax 39.8°C), accompanied by diplopia, bilateral ocular redness with discharge, and progressive periorbital edema. Outpatient treatment with ceftriaxone for one day, followed by ertapenem for one day, was ineffective. Initial outside imaging suggested sinusitis and cerebral ischemic lesions. On admission (August 19, Day 1), she was lethargic with marked inflammatory markers (white blood cell count [WBC] 18.06 × 10^9^/L, C-reactive protein [CRP] 98.3 mg/L, erythrocyte sedimentation rate [ESR] 102 mm/h (Table 1), D-dimer 786 ng/mL) and type I respiratory failure (PaO₂ 52 mmHg). The patient’s Clinical timeline and key interventions is summarized in Fig. 1, which provides a chronological overview of diagnostic milestones, therapeutic interventions, and key clinical events.

**Table 1:** Serial laboratory findings: complete blood count and inflammatory markers

Date	WBC (×10^9^/L)	NE (×10^9^/L)	HB (g/L)	PLT (×10^9^/L)	CRP (mg/L)	ESR (mm/h)	PCT (μg/L)	FER (μg/L)	D-D (μg/L)
2025/8/19	18.06	14.82	108	213	98.3	102	1.05	4123	786
2025/8/25	10.39	7.55	96	424	7.6	108	0.174	–	309
2025/8/29	9.09	7.41	93	351	0.8	73	–	1331	196
2025/9/4	4.06	3.26	93	154	0.6	103	0.101	1291	309
2025/9/7	4.67	2.94	89	131	–	91	–	1189	158
2025/9/28	4.96	3.28	82	162	<0.5	55	–	–	116
2025/10/2	3.01	1.70	73	112	<0.5	58	–	–	152
2025/10/5	3.34	2.02	68	102	<0.5	50	–	–	82
2025/10/8	4.86	2.96	66	150	<0.5	–	–	–	77

Abbreviations: WBC, white blood cell count; NE, neutrophil count; HB, hemoglobin; PLT, platelet count; CRP, Creactive protein; ESR, erythrocyte sedimentation rate; PCT, procalcitonin; FER, ferritin; DD, Ddimer; –, not tested

### Initial diagnostic workup

**Fig. 2:**
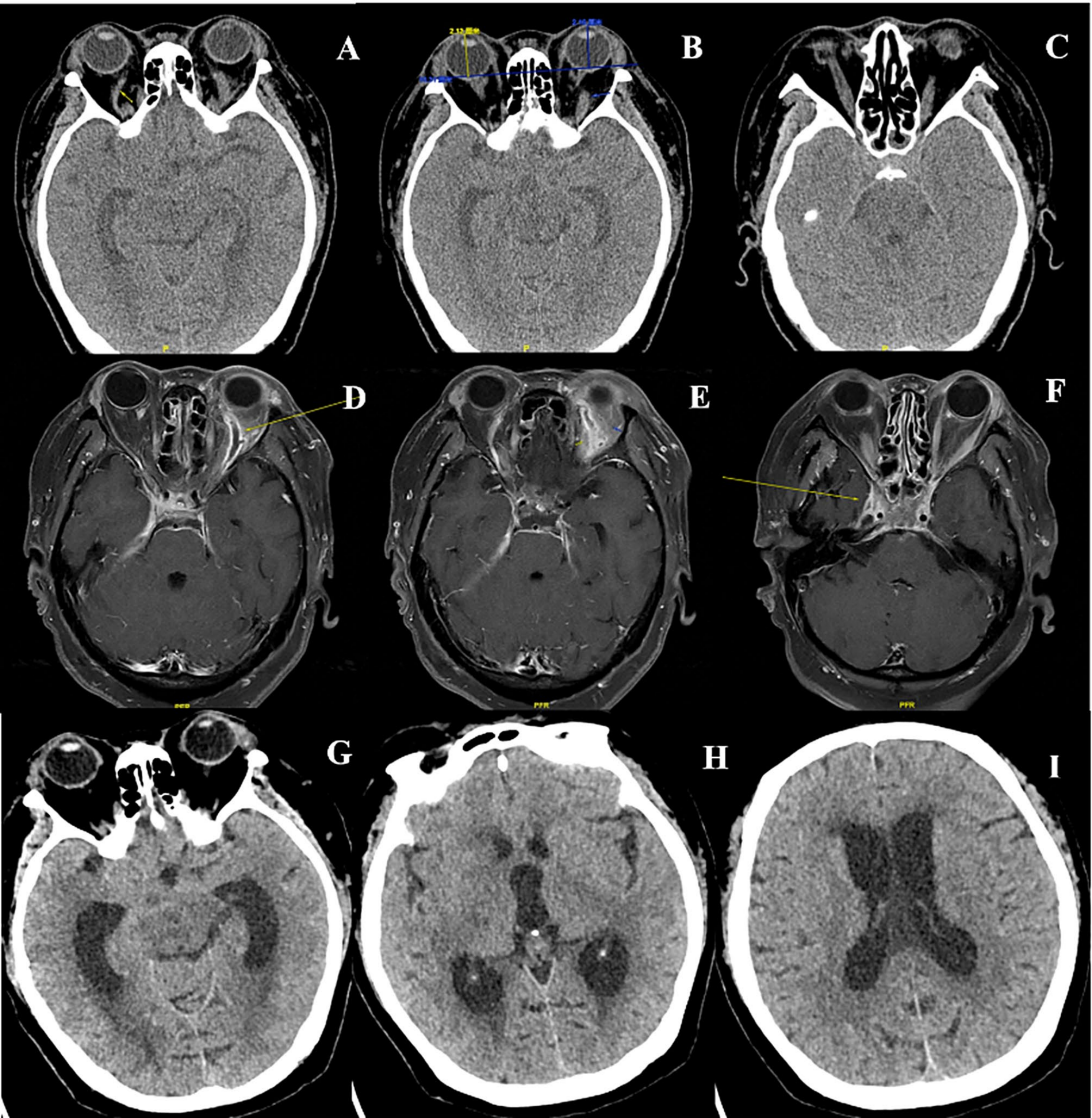
Serial neuroimaging findings. (**A**–**C**) orbital CT (August 23): bilateral proptosis and periorbital edema, dilation of the superior ophthalmic veins, and inflammatory opacification of the paranasal sinuses. (**D**–**F**) orbital MRI (August 23): confirms bilateral proptosis and dilated superior ophthalmic veins, suggestive of thrombophlebitis. (**G**–**I**) cranial CT (September 11): marked enlargement of the third and bilateral lateral ventricles, consistent with hydrocephalus; no evidence of hemorrhage

**Fig. 3:**
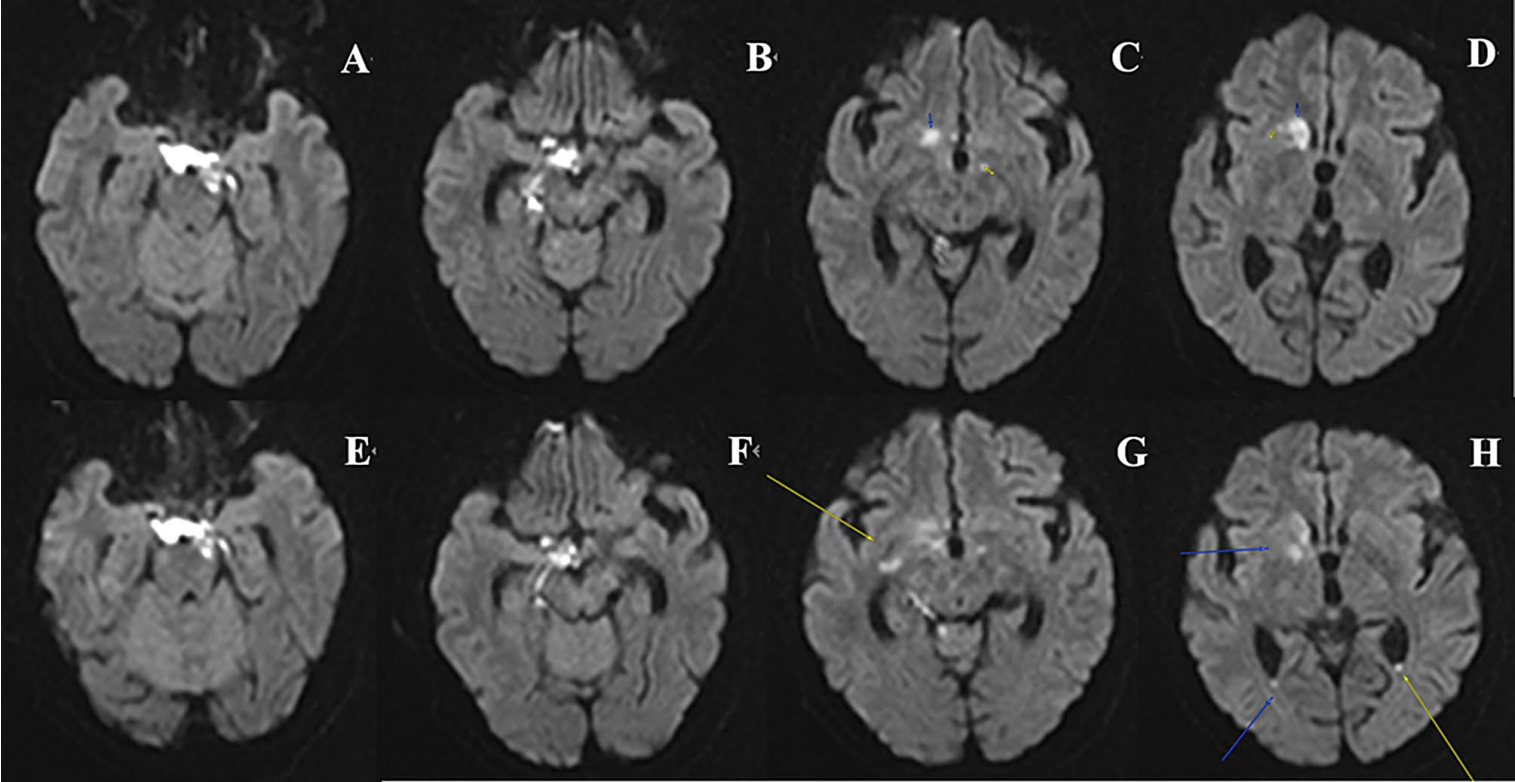
Brain MRI evolution. (**A**–**D**) Contrast-enhanced MRI (August 23): abnormal signal in the ambient and suprasellar cisterns, consistent with inflammation/early abscess formation. Right cavernous sinus widening with heterogeneous enhancement, suggestive of thrombosis. Diffusion-weighted imaging (DWI) shows hyperintense lesions in bilateral basal ganglia. (**E**–**H**) Follow-up MRI (August 26): interval increase in the number and extent of DWI hyperintense lesions within the bilateral basal ganglia, consistent with progressive cerebral infarction

**Fig. 4:**
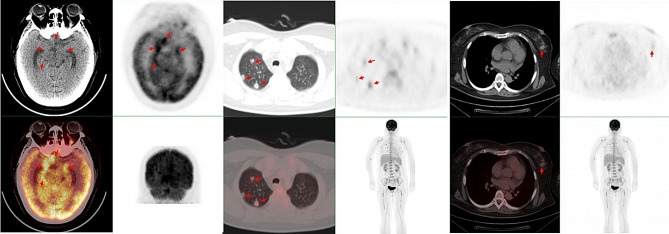
PET/CT findings. Multiple hypermetabolic foci are present in the brain parenchyma, suggestive of infectious or inflammatory processes. Bilateral pulmonary nodules also demonstrate increased FDG uptake, consistent with probable infectious etiology. The study further reveals metabolic activity in the paranasal sinuses, compatible with sinusitis. A left breast nodule shows low-grade FDG avidity, likely benign without features indicating malignancy

Dedicated orbital computed tomography (CT) and magnetic resonance imaging (MRI) (Fig. 2A–F) confirmed bilateral proptosis, dilated superior ophthalmic veins, and right cavernous sinus enlargement with heterogeneous enhancement—establishing the diagnosis of CST. Concurrent brain MRI revealed abnormal signal in the basal ganglia and suprasellar cisterns, consistent with early abscess formation or cerebritis (Fig. 3A–D). Cerebrospinal fluid (CSF) analysis via lumbar puncture on August 27 demonstrated pleocytosis (97 cells/μL; 91% mononuclear), hypoglycorrhachia (glucose 2.99 mmol/L), and elevated protein (0.88 g/L). Metagenomic next-generation sequencing (mNGS) of CSF detected *Staphylococcus aureus* (1 sequence read) (Table 2). Blood cultures and other microbiological studies (G test, GM test) were negative. Whole-body PET/CT (Fig. 4) showed multiple hypermetabolic foci in the brain and lungs, compatible with infectious processes, along with paranasal sinusitis; a left breast nodule showed low FDG uptake, likely benign.

**Table 2:** Serial cerebrospinal fluid (CSF) analysis and microbiological findings

Date	WBC [0–8]	RBC	Glucose (mmol/L) [2.5–4.5]	Protein (g/L) [0.15–0.45]	Chloride (mmol/L) [120–132]	Pathogen Identified
2025/8/27*	97	0	2.99	0.88	118	*Staphylococcus aureus* (mNGS)
2025/9/19	63	13	3.64	0.53	119	No growth
2025/9/23	44	110	3.63	0.97	122.8	Methicillin-resistant *S. aureus* (MRSA)
2025/9/26	0	30	3.54	0.7	121	MRSA
2025/9/29	8	130	4.32	0.49	122.9	MRSA
2025/10/3	3	35	3.27	0.51	122.2	*Pantoea agglomerans*
2025/10/7	4	0	3.85	0.42	125.3	Methicillin-resistant *Staphylococcus epidermidis* (MRSE)
2025/10/10	3	0	3.89	0.36	128.6	MRSA
2025/10/14	2	0	3.94	0.32	129.5	*Streptococcus salivarius*
2025/10/15*	7	0	3.23	0.68	125.5	*Bacillus subtilis*
2025/10/18	3	0	3.88	0.33	127.4	*Streptococcus sanguinis*

Footnotes: WBC, white blood cell count; RBC, redblood cell count; mNGS, metagenomic next-generationsequencing; MRSA, methicillin-resistant Staphylococcusaureus; MRSE, methicillin-resistant Staphylococcus epidermidis.“*” indicates results from lumbar puncture CSFspecimens; all other data represent CSF samples obtainedfrom the external ventricular drain.

### Clinical course and management

Following confirmation of CST, a multidisciplinary team (MDT) involving infectious diseases, neurosurgery, ophthalmology, and otorhinolaryngology was convened to guide management. Empiric therapy with meropenem (2 g every 8 hours) was initiated on Day 1 (Fig. 1). Following CSF mNGS results (Table 2), vancomycin (1 g every 12 hours) was added from Day 4–9. Adjunctive treatments included low-molecular-weight heparin (enoxaparin) for CST (see *Anticoagulation management* below) and a short course of methylprednisolone (40 mg daily, Day 6–10) and mannitol (Day 5–9) (Fig. 1).

Despite achieving therapeutic vancomycin trough levels (23.12 μg/mL), the patient developed new left limb weakness and dysarthria on Day 8. MRI showed progression of diffusion-restricting lesions in the basal ganglia, consistent with cerebral infarction possibly from septic emboli (Fig. 3E–H). We organized another MDT meeting, and therapy was switched to linezolid (600 mg every 12 hours) on Day 9 to improve central nervous system (CNS) penetration (Fig. 1).

On Day 24–25, she developed hallucinations, memory loss, urinary and fecal incontinence, and gait instability. CT on September 11 revealed marked ventricular enlargement indicative of obstructive hydrocephalus (Fig. 2G–I), without evidence of hemorrhage. Given this situation, another MDT meeting, comprising specialists from Neurology, Neurosurgery, and Psychology, was convened, and a surgical intervention for the hydrocephalus was subsequently decided upon. On Day 32 (September 19), she underwent Ommaya reservoir placement and external ventricular drainage. (Fig. 1). Postoperatively, neurological function improved. While systemic inflammatory markers normalized (Table 1), CSF cultures obtained from the ventricular drain remained persistently positive (Table 2).

### Anticoagulation management

Anticoagulation was initiated on Day 5 (August 23) with enoxaparin 4000 IU once daily following CST diagnosis. Although concurrent brain abscesses raised concerns about hemorrhagic risk, the benefits of anticoagulation for septic thrombosis were deemed to outweigh potential complications; initial D-dimer was only mildly elevated (786 ng/mL). Following clinical deterioration suspicious for septic emboli on Day 8—with no imaging evidence of hemorrhage—anticoagulation was intensified to therapeutic-dose enoxaparin (4000 IU twice daily) from August 28 to September 18 (Fig. 1). Anticoagulation was temporarily discontinued on September 18 in preparation for Ommaya reservoir placement. Postoperatively, the presence of hemorrhagic CSF (elevated red blood cell counts in drainage) precluded resumption of anticoagulation for the remainder of the hospital course.

### Intraventricular vancomycin therapy

Following transfer to a tertiary neurosurgical center on October 23 (Day 66), the patient received intraventricular vancomycin via the external ventricular drain. The regimen was individualized based on ventricular size and daily CSF drainage volume. Given significant ventriculomegaly, vancomycin 15 mg (diluted in preservative-free normal saline) was administered once daily. The dosing interval was determined by 24-hour CSF drainage volume (approximately 150–200 mL/day), which supported once-daily administration to maintain therapeutic levels. The drain was clamped for 60 minutes following each dose to allow drug distribution. Serial CSF cultures obtained on October 30, November 3, November 5, November 7, November 10, and November 13 were all negative. A targeted next-generation sequencing (tNGS) assay performed on November 12 was also negative, confirming microbiological eradication (Fig. 1). No adverse events attributable to intraventricular therapy—such as chemical ventriculitis or seizures—were observed. A permanent ventriculoperitoneal shunt was successfully placed on December 11 (Day 115), with sustained clinical improvement at follow-up.

At the most recent follow-up in March 5, 2026, the patient had achieved complete neurological recovery. The previously documented abducens nerve palsy had fully resolved, and she was living independently without any significant neurological deficits.

## Discussion

Despite the well-documented nature of CST in the literature, this case offers several distinctive contributions that extend beyond mere description. **First, it illustrates a protracted and refractory disease course**—characterized by persistent infection despite appropriate systemic antibiotics and the development of secondary intracranial complications. Such a trajectory provides a valuable educational framework for clinicians confronting treatment failure in complex CNS infections.

**Second, this report provides granular, real-world data on intraventricular vancomycin therapy—an intervention often mentioned but rarely detailed in the literature.** We present a comprehensive account of our dosing rationale (15 mg daily), administration technique (60-minute drain clamping), and safety outcomes. These practical details address a critical knowledge gap and offer a template for clinicians considering direct CNS antimicrobial delivery in refractory cases.

**Third, the case underscores the indispensable role of a multidisciplinary team (MDT) in navigating diagnostic ambiguity, timing of surgical interventions, and dynamic treatment adjustments.** Serial MDT discussions facilitated early recognition of complications and guided nuanced perioperative management. This collaborative framework was the cornerstone of achieving a favorable outcome.

### Diagnostic challenges in an atypical host

Diagnostic delay stemmed from non-specific symptoms mimicking orbital cellulitis [4, 7, 8], absence of typical facial sources, and inconclusive early imaging. The key clue was concurrent altered mental status with painful ophthalmoplegia, confirmed by dedicated orbital MRI showing superior ophthalmic vein dilation [9]. Her immunocompromised state (diabetes, alcohol use)—risk factors in over one-third of CST patients [2, 3]—should lower diagnostic threshold.

The patient initially presented with diplopia, blurred vision, binocular pain (more severe on the right), and limited abduction of the right eye. She was diagnosed with “ophthalmoplegia” at an outside hospital and received subcutaneous injections of compound anisodine hydrobromide in the right temporal region, adjacent to the superficial temporal artery, for two consecutive days, two days before the onset of systemic symptoms. However, her ocular symptoms did not improve. Given the absence of typical facial sources of infection (e.g., sinusitis, dental foci), this iatrogenic intervention was considered a potential portal of entry for MRSA, particularly in the context of poorly controlled diabetes. This history highlights the importance of obtaining detailed procedural and injection histories in patients with cryptic S. aureus infections, especially when classic facial sources are absent.

### Microbiological interpretation: infection vs. contamination

Serial CSF cultures posed recurring diagnostic dilemmas [10]. Initial lumbar puncture CSF grew *S. aureus* with pleocytosis, establishing the primary pathogen. Post-Ommaya, three consecutive MRSA cultures with persistent mild CSF abnormalities (WBC 44–63/μL, protein 0.53–0.97 g/L) suggested true infection, warranting continued anti-MRSA therapy. However, subsequent isolates—Pantoea agglomerans, methicillin-resistant Staphylococcus epidermidis (MRSE), Streptococcus salivarius, and Streptococcus sanguinis—were each recovered only once, accompanied by normalizing CSF parameters (WBC < 5/μL, glucose > 3.5 mmol/L, protein < 0.45 g/L) and complete clinical stability (afebrile, no new neurological deficits, with peripheral white blood cell count and neutrophil count not elevated in the absence of systemic infection, and C-reactive protein remaining at low/normal levels). The transient appearance of these low-virulence organisms, commonly regarded as skin or environmental contaminants, together with the absence of sustained CSF pleocytosis or clinical deterioration, pointed toward contamination rather than true EVD-associated ventriculitis. This interpretation was corroborated by a confirmatory lumbar puncture on October 15, which grew only Bacillus subtilis—another likely contaminant—while CSF indices remained normal. Additionally, targeted next-generation sequencing (tNGS) of ventricular CSF on November 12 was negative for all pathogens, further supporting the conclusion that prior positive cultures did not represent active infection. This stepwise, multimodal approach—integrating clinical course, serial CSF parameters, pathogen characteristics, and molecular diagnostics—enabled appropriate narrowing of antimicrobial therapy and avoided unnecessary treatment extension.

### Overcoming the blood-brain barrier

Despite therapeutic vancomycin troughs and subsequent linezolid, infection progressed with new septic emboli—underscoring the limitations of systemic therapy in CNS infections. Definitive CSF sterilization occurred only after intraventricular vancomycin, directly bypassing the blood-brain barrier. Our regimen followed established guidelines [11]: 15 mg daily (adjusted for ventriculomegaly), with dosing interval determined by CSF drainage (150–200 mL/day supporting once-daily administration). No neurotoxicity was observed. This supports proactive consideration of intrathecal therapy in refractory, culture-positive cases [12].

### Anticoagulation in complex CST

Anticoagulation was initiated cautiously (enoxaparin 4000 IU daily) given concurrent brain abscesses and mild D-dimer elevation (786 ng/mL). Following clinical deterioration suspicious for septic emboli, therapy was intensified to therapeutic dosing (4000 IU twice daily) from August 28 to September 18. During this period, to monitor for hemorrhagic transformation, we performed daily neurological assessments and serial non-contrast head CT scans as clinically indicated. The patient’s neurological status remained stable, and serial laboratory tests showed stable hemoglobin and platelet counts, as well as no evidence of coagulation abnormalities. No clinical or radiological evidence of intracranial hemorrhage was observed. Anticoagulation was temporarily discontinued on September 18 in preparation for Ommaya reservoir placement. Postoperatively, the presence of hemorrhagic CSF (elevated red blood cell counts in drainage) precluded resumption of anticoagulation for the remainder of the hospital course. While meta-analyses support mortality benefit with anticoagulation in CST [13], our experience highlights the need for individualized risk assessment when intracranial complications coexist.

### The central role of multidisciplinary collaboration

Optimal outcome was contingent upon seamless MDT integration (infectious diseases, neurosurgery, ophthalmology, ENT). Serial MDT discussions enabled timely complication recognition (cerebral infarction, hydrocephalus), informed escalation to intraventricular therapy, and guided nuanced perioperative management. This collaborative framework, rather than any single intervention, was the cornerstone of source control [6, 14].

### Long-term outcome and clinical implications

The patient’s complete neurological recovery at the most recent follow-up (March 2026) further validates the effectiveness of the multidisciplinary treatment strategy, particularly the role of intraventricular vancomycin in achieving source control. The absence of residual deficits underscores the potential for full functional recovery even in refractory CST with severe intracranial complications, provided timely and aggressive intervention is pursued.

This case yields several practical insights. First, it reinforces that diabetes and chronic alcohol use are significant risk modifiers that should lower the threshold for investigating CST. Second, it demonstrates that intraventricular antibiotic therapy can be decisive in overcoming pharmacokinetic barriers in refractory cases, and provides a practical dosing template. Third, it validates a structured MDT framework as the standard of care for managing CST with neurological complications.

Our report has limitations inherent to a single case study. The precise origin of infection (iatrogenic vs. subclinical sinusitis) remains undetermined. While managed effectively, the potential for the indwelling Ommaya reservoir to contribute to biofilm-related persistence cannot be entirely excluded. Additionally, key follow-up laboratory data (including the CSF tNGS result from November 12, 2025) were obtained from an external institution, limiting our ability to independently verify the original data and detailed methodological parameters.

## Conclusion

This refractory case of MRSA-associated CST with intracranial complications illustrates that successful outcomes depend on: (1) early recognition in at-risk hosts, (2) decisive escalation to direct CNS drug delivery when systemic therapy fails—with a practical, evidence-based regimen for intraventricular vancomycin, and (3) seamless integration of multidisciplinary expertise from the outset. The detailed dosing and monitoring data provided here address a knowledge gap and offer a clinical template for managing similar complex, multidrug-resistant CNS infections, with the potential for complete neurological recovery as demonstrated in this case.

## Data Availability

The raw metagenomic sequencing data generated during this study are available in the National Genomics Data Center (NGDC) Genome Sequence Archive (GSA) repository under BioProject accession number PRJCA055011 (https://ngdc.cncb.ac.cn/bioproject/browse/PRJCA055011). The data are currently under private access and will be made publicly available upon publication of this article. All other clinical data (including medical records and imaging studies) supporting the findings of this study are available within the article, or from the corresponding author on reasonable request, subject to privacy and ethical restrictions.

## Abbreviations

CST: Cavernous sinus thrombophlebitis
MRSA: methicillin-resistant Staphylococcus aureus
mNGS: Metagenomic next-generation sequencing
CSF: cerebrospinal fluid
MDROs: multidrug-resistant organisms
WBC: white blood cell count
NE: neutrophil count
HB: hemoglobin
PLT: platelet count
CRP: Creactive protein
ESR: erythrocyte sedimentation rate
PCT: procalcitonin
FER: ferritin
DD: D dimer
CNS: central nervous system
TDM: therapeutic drug monitoring
tNGS: targeted next-generation sequencing
SOV: superior ophthalmic vein
EVD: external ventricular drain
VP: ventriculoperitoneal
MDT: multidisciplinary team
